# Mechanical Characterizations of 3D-printed PLLA/Steel Particle Composites

**DOI:** 10.3390/ma12010001

**Published:** 2018-12-20

**Authors:** Hozhabr Mozafari, Pengfei Dong, Haitham Hadidi, Michael P. Sealy, Linxia Gu

**Affiliations:** Department of Mechanical & Materials Engineering, University of Nebraska-Lincoln, Lincoln, NE 68588, USA; hozhabr.mozafari@huskers.unl.edu (H.M.); donphy@huskers.unl.edu (P.D.); haitham.hadidi@huskers.unl.edu (H.H.); sealy@unl.edu (M.P.S.)

**Keywords:** particle composite, 3D printing, fused filament fabrication, finite element method, micromechanics, poly-l-lactic acid (PLLA), nanoindentation, mechanical properties

## Abstract

The objective of this study is to characterize the micromechanical properties of poly-l-lactic acid (PLLA) composites reinforced by grade 420 stainless steel (SS) particles with a specific focus on the interphase properties. The specimens were manufactured using 3D printing techniques due to its many benefits, including high accuracy, cost effectiveness and customized geometry. The adopted fused filament fabrication resulted in a thin interphase layer with an average thickness of 3 µm. The mechanical properties of each phase, as well as the interphase, were characterized by nanoindentation tests. The effect of matrix degradation, i.e., imperfect bonding, on the elastic modulus of the composite was further examined by a representative volume element (RVE) model. The results showed that the interphase layer provided a smooth transition of elastic modulus from steel particles to the polymeric matrix. A 10% volume fraction of steel particles could enhance the elastic modulus of PLLA polymer by 31%. In addition, steel particles took 37% to 59% of the applied load with respect to the particle volume fraction. We found that degradation of the interphase reduced the elastic modulus of the composite by 70% and 7% under tensile and compressive loads, respectively. The shear modulus of the composite with 10% particles decreased by 36%, i.e., lower than pure PLLA, when debonding occurred.

## 1. Introduction

Three-dimensional printing is gaining increased attention due to the fabricating of complex composite polymer structures with minimal waste of raw materials. Printed polymer composites reinforced by fibers and particles have been considered to overcome the limited strength and functionality of the pure polymer [1]. For example, metallic particles reinforce 3D printed polymer composites in terms of mechanical, thermal, and electrical properties. Nikzad et al. [2] developed iron/acrylonitrile butadiene styrene (ABS) and copper/ABS 3D printed particle composites to achieve higher stiffness. Boparai et al. [3] examined tribological characteristics of a composite material with Al and Al_2_O_3_ particles embedded in a nylon 6 matrix. As a result, higher wear resistance, thermal stability, and stiffness were attained. Bedi et al. [4] studied the effect of reinforcing Low-density polyethylene (LDPE) polymer with SiC/Al_2_O_3_ particles. They found that the Al_2_O_3_ reinforcement of LDPE resulted in better dimensional stability with improved surface hardness. Impact resistance, tensile strength, and electromagnetic characterization of a 3D printed tungsten–polycarbonate polymer matrix composite was experimentally investigated for space-based applications [5]. Fracture behavior and interlayer bonding of 3D printed carbon fiber and glass fiber reinforced thermoplastics has been evaluated [6,7]. In addition, the interphase properties and interface strength play important roles in the mechanical properties of composites [8]. Existing research focused on the effect of particle size [9], distribution [10], and shape [11] on the mechanical properties of composites; however, the contribution of the interphase was less addressed, especially for 3D printed particle composites. The existence of an interphase is mainly due to the inhomogeneity of the material, i.e., the high stiffness ratio between particles and matrix [12]. Analytical models were developed to encompass the influence of the interphase layer. The earliest models [13] assumed that the two components were both homogeneous and were perfectly bonded across a sharp and distinct interface. Hashin and Rosen [14] developed a model for composites in which a thin layer existed outside of each particle. The elastic moduli were uniform within this layer, but different from those in the matrix or particles. Others have attempted to account for smooth variation of the moduli in response to the distance from the particle. Lutz and Zimmerman [15] modeled the moduli outside of the inclusion with a constant term plus a power-law term, thereby allowing a smooth transition between the interphase layer and the matrix. Despite the capability of analytical models to calculate bulk properties of composites, determining the local microstructure parameters, such as the effective interphase thickness and fluctuations of the elastic modulus, is not feasible by this approach. Therefore, nanoindentation has become the standard technique for characterization of local properties of materials.

Nanoindentation measurement has a broad application across the physical sciences [16]. The material properties of the interphase in phenolic/glass and polyester/glass systems were quantified by Hodzic et al. [17]. The mechanical property variations were observed within the interphase of E-glass fiber reinforced epoxy resin and E-glass fiber reinforced modified polypropylene (PPm) matrix composites [18]. Urena et al. [19] measured the mechanical properties of the interphase by reaction between the aluminum matrix and SiO_2_ coating. Hardness and Young’s modulus of the interphase between matrix and reinforcement of Al 2014 matrix composites reinforced with (Ni_3_Al)*_p_* were determined by Torralba et al. [20]. These studies have provided valuable information about the formation and characterization of particle composites. Chacon et al. characterized the effect of build orientation, layer thickness and feed rate on the mechanical performance of a pure poly lactic acid (PLA) polymer [21]. However, the studies on reinforced poly-l-lactic acid (PLLA) composites are scarce [22].

In this study, mechanical properties of 3D printed PLLA reinforced by grade 420 stainless steel (SS) particles were characterized by nanoindentation tests with a specific focus on the interphase. Fused filament fabrication was employed to manufacture PLLA/420SS composite specimens. Nanoindentation tests were performed to quantify the Young’s modulus of the matrix, particles, and the interphase layer. The bulk mechanical properties of the PLLA/420SS composite were calculated through a micro-scale representative volume element (RVE). Load sharing of the steel particles versus the particle’s volume fraction has been obtained. Moreover, the influence of interface deterioration on the macro-mechanical properties of the composite was identified with respect to three different loading scenarios.

## 2. Materials and Methods

### 2.1. 3D Sample Fabrication

**Figure 1:**
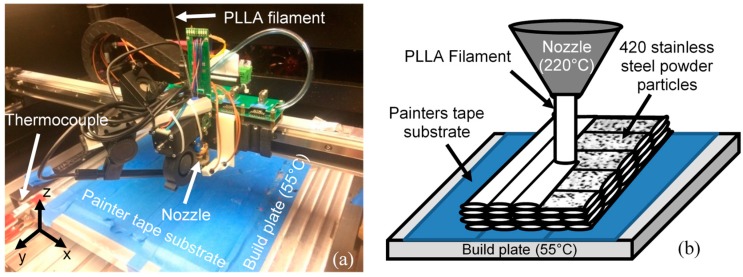
Equipment and schematic of 3D printing poly-l-lactic acid (PLLA) Steel composite, (**a**) fused filament fabrication (FFF) Hyrel Hydra 645 3D printer, (**b**) FFF process schematic.

Additive manufacturing (AM) refers to a group of fabrication in which parts are fabricated layer by layer [23]. This manufacturing method has drawn the attention of researchers in high-tech industries such as aerospace and unmanned aerial vehicle (UAVs) design [24]. Fused filament fabrication, also known as fused deposition modeling (FDM), is one of the most commonly used additive manufacturing (AM) technologies. The composite samples were printed by fused filament fabrication on a Hyrel Hydra 645. A continuous filament of amorphous thermoplastic material is extruded through a heated nozzle and deposited in a single track. Typically, a raster pattern is used to form a single layer by moving the printing head (nozzle) horizontally (*x*-*y*). Deposited material promptly solidifies and adheres with adjacent tracks of material to form the required geometry; see Figure 1.

The process is repeated as the platform moves vertically (*z* direction) to enable deposition of another layer. Commonly used materials for FFF include acrylonitrile butadiene styrene (ABS) and PLLA. One of the main advantages of AM compared to other traditional manufacturing processes is to access each layer for modifying material properties.

**Figure 2:**
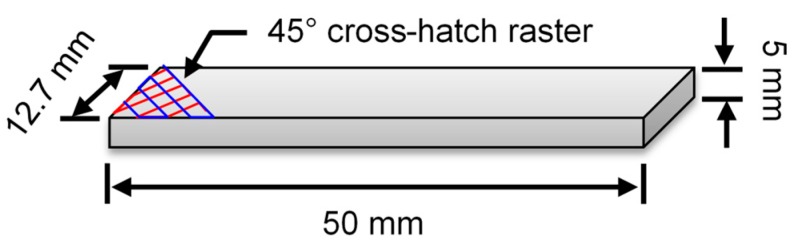
3D printed PLLA-SS420 composites.

The physical and mechanical properties of PLLA and 420SS are provided in Table 1. The PLLA filament from 3D4MAKERS (Haarlem, The Netherlands) had a natural color and diameter of 1.75 mm. Micro-melt 420LC stainless steel powder from carpenter powder products had a diameter between 45 and 105 µm. A total of 25 layers were printed for each sample at a layer thickness of 0.2 mm, 90% infill density, and a rectilinear infill pattern with a 45° infill angle. At 220 °C, the nozzle temperature was above the glass transition temperature of PLLA and allowed for smooth material flow for deposition. The build plate was heated to 55 °C to promote adhesion with the part. The travel speed of the nozzle was 50 mm·s^−1^. The dimensions of each sample were 50 mm × 12.7 mm × 5 mm (Figure 2). A brim of 4 mm was also printed to help secure the edges of the part to prevent warping and improve layer adhesion to the build platform.

### 2.2. Nano-Indentation Tests

**Figure 3:**
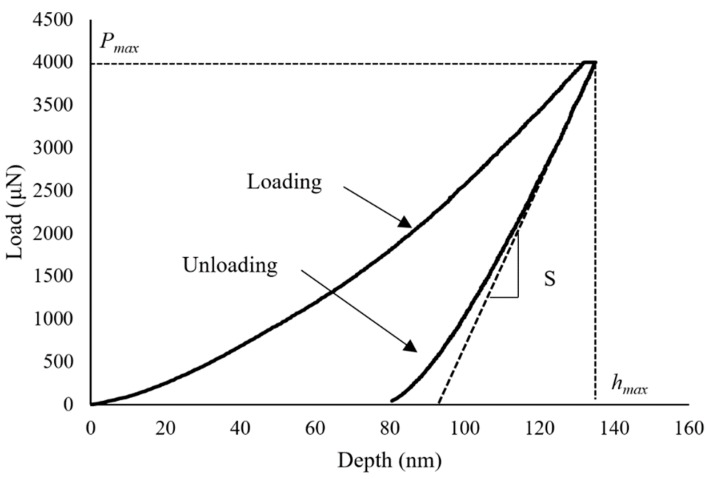
Load-depth curve for the nano-indentation test for the steel particles.

The nanoindentation test was conducted using the commercial Hysitron TI 950 TriboIndenter (Billerica, MA, USA) [25]. The force–displacement curve during loading and unloading was recorded for calculating mechanical properties including the elastic modulus [26]. A representative load-depth curve for both loading and unloading for the nano-indentation of the steel particles has been depicted in Figure 3.

In our experiment, a three-sided pyramidal cube corner probe was used. This probe has a half angle of θ = 35.26°, an aspect ratio of 1:1, and an average radius of curvature of 100 nm. The indentation depth *h*_c_ and projected contact area *A* were calculated based on the geometric information of the probe [27,28]:

(1) $$h_{c}=h_{\max}-\omega \frac{P_{\max}}{S}$$

(2) $$A=3\sqrt{3}h_{c}^{2}\tan^{2}\theta =3\sqrt{3}h_{c}^{2}\tan^{2}\left(35.26\right)=2.60h_{c}^{2}$$

In which, *h*_max_, *P*_max_, *S* represent the maximum displacement of the indenter, maximum load, and the slope of the unload curve at the *P*_max_, which is ($\frac{dP}{dh}){|}_{h=h_{\max}}$, respectively. In addition, ω is 0.75 for the cube corner probe. Based on the contact depth *h*_c_ and the projected contact area *A*, the calculated elastic modulus E* is:

(3) $$E^{*}=\frac{1}{2}\frac{\sqrt{\pi }}{\sqrt{A}}S=\frac{1}{2}\frac{\sqrt{\pi }}{\sqrt{A}}\frac{dP}{dh}$$

### 2.3. Finite Element Modeling

**Figure 4:**
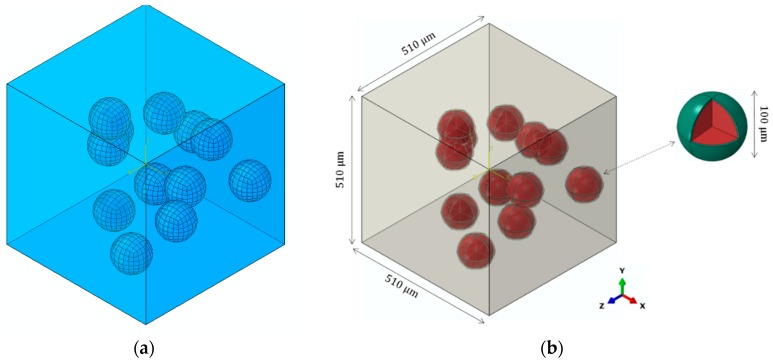
(**a**) The generated representative volume element (RVE) and (**b**) dimensions.

A micromechanical representative volume element (RVE) model was created by using the Digimat software version 4.2.1 (e-Xstream Engineering, Hautcharage, Luxembourg), as shown in Figure 4. Digimat has the capacity to construct microscopic configurations of composite materials and to derive micromechanical material models suited for coupling with FE software ABAQUS (2016). The steel particles were randomly distributed in the PLLA polymer matrix. Steel particle size was defined in accordance with our experimental measurements. The selection of RVE size was based on the following two criteria [29]:

1. An RVE should be sufficiently large to include the essential microstructural characteristics.
2. The RVE size should be as small as possible so that the states of stress and strain can be homogenized in the whole model.

After initial analyses, the optimum RVE size was detected to be 510 µm. Perfect bonding was considered at the interface of particles and interphase by using a surface-based tie constraint, which is used to make the translational and rotational motion as well as all other active degrees of freedom equal for a pair of surfaces. Moreover, it allows for rapid transitions in mesh density between components. To study the effect of bonding strength, imperfect bonding was modeled by replacing the tie constraint with a surface-based contact constraint between the interphase and matrix. Friction coefficient of 0.3 was applied; therefore, the interacting surfaces can undergo either small or finite sliding [30].

The volume fraction of particles was set to 3%, 5%, and 10% to evaluate the load-sharing effect of steel particles inside the PLLA polymeric matrix. Mesh sensitivity analysis was performed and the element size in range of 0.0072 to 0.04 µm was chosen. The model was meshed with C3D8R elements and the number of elements varied from approximately 89,000 to 251,000 depending on the volume fraction of particles. A periodic boundary condition, developed using a user-defined Python script, was enforced in all directions to extend the RVE periodically, i.e., to consider the interaction between the RVE and its mirrored images. The periodic boundary condition was expressed in terms of the displacement vector *u*, which related the displacements between the opposite edges according to

(4) $$u\left(x,y,0\right)-u_{z}=u\left(x,y,L\right)\;u\left(x,0,z\right)-u_{y}=u\left(x,L,z\right)\;u\left(0,y,z\right)-u_{x}=u\left(L,y,z\right)$$

where *L* was the length; *x*, *y*, and *z* stood for the coordinate axes of the three edges of the RVE; *u_x_*, *u_y_* and *u_z_* depended on the particular loading applied to the RVE. Three loading scenarios are considered in this study: Tension, compression and shear in *X*-*Y* plane. The load shared by each phase was calculated as the integration of all nodal forces along the loading direction.

## 3. Results and Discussion

### 3.1. Nanoindentation Tests

**Figure 5:**
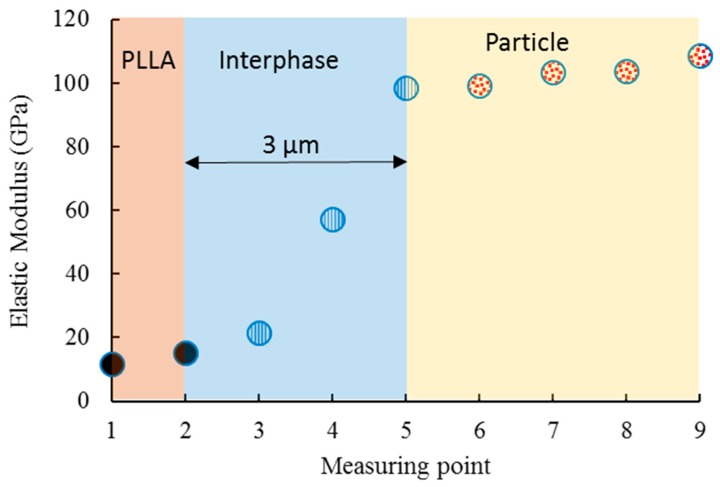
Nanoindentation response at a region close to the particle’s edge.

The obtained elastic modulus across three phases of the composite was illustrated in Figure 5. The variation of elastic modulus values from the PLLA toward the steel particle was observable. Hereby, we examined the interface and interphase properties.

The interface is defined as the two-dimensional boundary between the matrix and the particles, while the interphase is the three-dimensional region that includes the interface plus a zone of finite thickness on both sides of the interface. The interphase boundaries are generally defined from the point in the matrix where the local properties start to deviate from the bulk properties in the direction of the polymer/particle interface.

The interphase layer, although with small absolute thickness, can gives graded properties to the composite. Therefore, the relative thickness of interphase layer was suggested [31] as the main parameter to cope with the contribution of interphase layer in mechanical properties of the composite. Since the softening effect around the particle edges is controlled by the relative thickness, it is preferable to have smaller relative thickness of interphase layer. Smaller softening zone will help to have more accurate estimation of elastic modulus of the composite [31]. The average thickness of the interphase was detected at approximately 3 µm, which is relatively small (approximately 3% of the particle diameter). This small relative thickness makes it easier to predict the mechanical properties of composite and decreases the possibility of existing defects associated with the bonding interface

**Figure 6:**
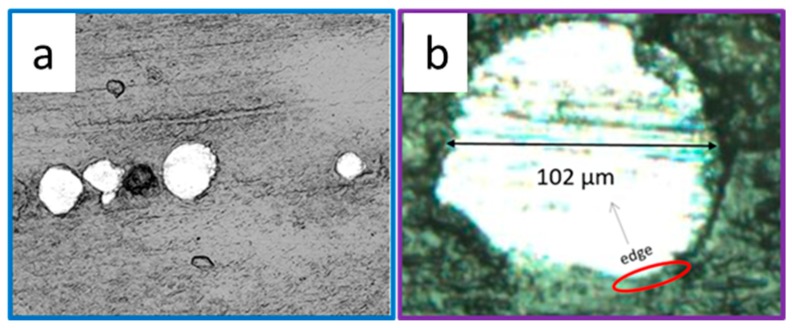
Scanning electron microscope (SEM) images of the composite (**a**) and its zoom-in view of the particle region (**b**).

Bonding characteristics of particles against the matrix is strongly related to the interface quality, which is depending on the manufacturing method and composite constituents. The scanning electron microscope (SEM) of the composite detected no obvious interface defects in the 3D printed specimen, as shown in Figure 6. This indicates good cohesive behavior between PLLA melt and 420SS particle under processing conditions employed in the current study.

### 3.2. Homogenization of the Poly-l-lactic Acid (PLLA) /Steel Composite

The macro-mechanical properties of the 3D-printed PLLA/420SS particle composite can be achieved by homogenization of the RVE model. After examining the elastic modulus of the composite phases, the obtained values were imported into the finite element model, and numerical simulations were conducted. It should be noted that the RVE model was established based on the previous validated modeling framework [32].

**Figure 7:**
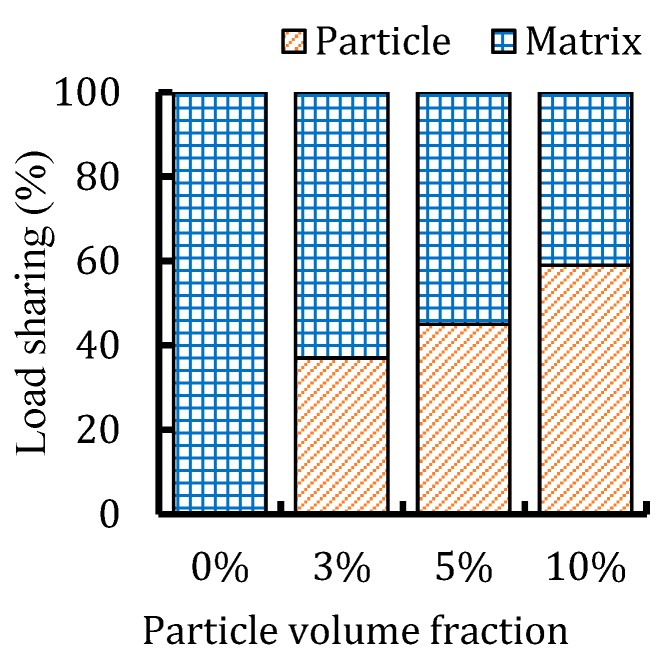
Load sharing capacity of the PLLA/420SS composites.

The role of particles in strengthening of the PLLA matrix can be demonstrated by comparing the load sharing of the composites. The load shared by each phase was calculated by the integration of all nodal forces along the loading direction (Figure 7). The results showed that the load shared by the steel particle increased by 21% when the volume fraction varied from 3% to 10%. The contribution of the interphase was insignificant, which was mainly attributed to the low thickness of this layer. The load sharing capacity is related to the mechanical properties, size, volume fraction, and shape of the particles embedded in the matrix.

The authors studied the reinforcing effect of Mg particles in PLLA matrix in their previous study [32]. Comparing the obtained results, it can be concluded that the load sharing capacity of 420SS particles are 10% to 15% higher than that for Mg particles for the identical volume fraction when embedded in PLLA polymer matrix. Higher stiffness of 420SS particles allow them to take more load when the composite exposed to external forces. 

**Figure 8:**
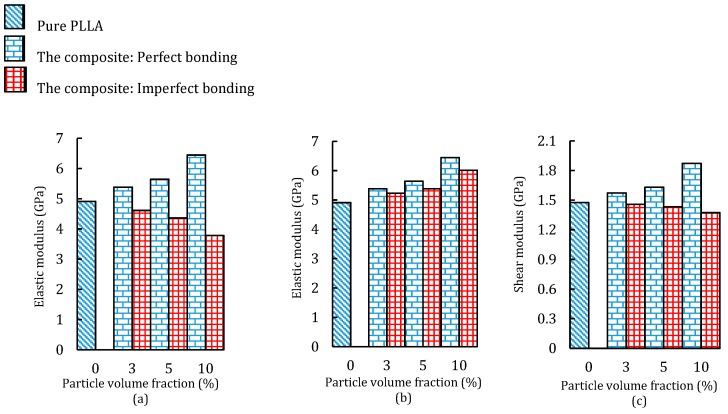
Effective elastic modulus of composite under (**a**) tensile, (**b**) compressive, and (**c**) shear loadings.

Degradation of the matrix after a while can cause deterioration of the interphase layer and then debonding of the particles [33]. Existence of interfacial debonding can decrease the strength of composites and initiate progressive internal damage [34]. Although, debonding of particles was not observed for the 3D printed composites, due to the degradability of the PLLA polymer over time, we expected to see decohesion of particles over time; therefore, we studied the effect of possible interfacial defects on the performance of 3D printed PLLA/420SS composite. Figure 8 shows the contribution of particle bonding strength in different loading scenarios. 

As the particle volume fraction increased to 10%, the effective elastic modulus of the composite increased by 31% (Figure 8a). This enhancement of effective elastic modulus is substantial when compared with our previous work [32] in which the Mg particles enhanced this parameter to 16%. Imperfectly bonded particles exhibited a much smaller elastic modulus, even lower than that for the pure PLLA polymer. This implied the importance of the fabrication process of composites. A comparison of the composite with 10% particles shows that bonding defects can reduce its elastic modulus by 70%, while this difference for PLLA/420SS with 5% and 3% particles is up to 29%, and 17%, respectively. This proves that when degradation of PLLA occurs, the composites with a higher initial elastic modulus will be the weakest ones, which can be accounted as a limitation for embedding a higher volume fraction of particles [35].

The obtained values of the elastic modulus under a uniaxial compressive load are depicted in Figure 8b. As was expected, imperfect bonding condition played a minimal role in the variation of the elastic modulus. Comparatively, all the PLLA/420SS composites had a higher elastic modulus than the pure PLLA. The largest reduction of elastic modulus was 7.2% for the composite with 10% particles. This result shows that when the 3D printed particle composite is exposed to the compressive loads, even when PLLA polymer degradation occurs, the sustainability of the structure is not affected largely. It can be seen that PLLA/420SS with 10% of particle fraction increased the shear modulus of the PLLA polymer up to 26% (Figure 8c). In addition, in the worst case, debonding of particles could decrease the shear modulus down to 36%.

**Figure 9:**
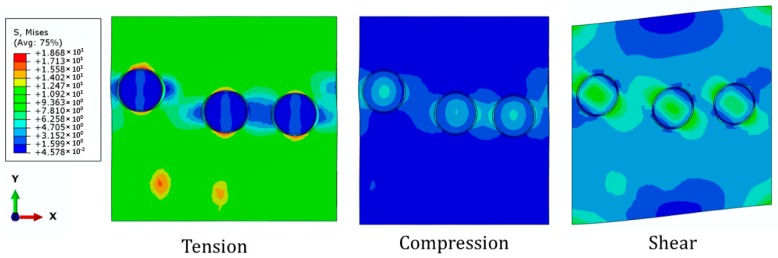
Stress distribution of PLLA/420SS composite with volume fraction of 3% under different loading scenarios (imperfect bonding).

The stress distributions of the PLLA/420SS composite with volume fraction of 3% subjected to the loading conditions are depicted in Figure 9. When the specimen is under tension in *Y* direction, there is a sharp decrease of stress around particles, perpendicular to the loading direction. This could be explained by the reduction of the composite’s integrity. On the other hand, the compression induced a smoother stress distribution among particles. Finally, for the shear deformation in *X*-*Y* plane, the debonding effect on the stress distribution was recognizable and decohesion of particles at the angle of 45° and 275° with respect to *x*-axis was observable.

The interface decohesion and fracture of the particle composite is more probable when the tensile load is dominant as illustrated in [36]. It has been observed that the plastic deformation of the matrix around the particles leads to the formation of a void in between the particle halves or at the poles. These voids then are the main cause of crack nucleation and fracture of the composite [36]. According to the experimental results by [37] fracture occurs suddenly by unstable crack propagation of a crack perpendicular to the tensile loading axis through the equator of particles and propagates gradually through the interface as the applied load increases. 

## 4. Conclusions

In this paper, a 3D printed PLLA/420SS particle composite was manufactured using the fused filament fabrication method. Due to the interesting properties of this composite, there could be many applications, including magnetic devices, and flexible electronics, especially pressure and strain sensors [34]. Nanoindentation tests were conducted in order to characterize the micro-mechanical properties of each composite and the thickness of the interphase. At the last step, an RVE model was generated to determine the elastic properties of the composite. The following conclusions can be extracted:

- The elastic modulus of a 3D printed composite with 10% particles was 31% higher than that for the pure PLLA polymer.
- Perfect bonding of particles was observed for almost all the samples.
- The measured thickness of the interphase was considerably lower than the diameter of the particles, and a sharp variation of the elastic modulus was observed around the particle’s edge.
- The sharing load of particles in the composite with 10% particles was approximately 60%, whereas this value for the composites with less than 10% particles was lower than 50%.
- Degradation of the interphase could reduce the elastic modulus of the composite by 70% and 7% under tensile and compressive loads, respectively.
- The shear modulus of the composite with 10% particles decreased by 36% when debonding occurred. In this case, the shear modulus of the composites was lower than that for the pure PLLA polymer.

## Figures and Tables

**Table 1:** Physical and Mechanical Properties of poly-l-lactic acid (PLLA) and 420 Stainless Steel.

Component	Density (g·cm^−3^)	Tensile Strength (MPa)	Flexural Modulus (GPa)
PLLA	1.26	71	3.31
420 stainless steel	7.72	1793	199.95
